# Decreased Inflammatory Responses of Human Lung Epithelial Cells after Ethanol Exposure Are Mimicked by Ethyl Pyruvate

**DOI:** 10.1155/2014/781519

**Published:** 2014-11-03

**Authors:** B. Relja, N. Omid, K. Kontradowitz, K. Jurida, E. Oppermann, P. Störmann, I. Werner, E. Juengel, C. Seebach, I. Marzi

**Affiliations:** ^1^Department of Trauma, Hand and Reconstructive Surgery, University Hospital Frankfurt, Goethe University, 60590 Frankfurt, Germany; ^2^Department of General Surgery, University Hospital Frankfurt, Goethe University, 60590 Frankfurt, Germany; ^3^Department of Thoracic and Cardiovascular Surgery, University Hospital Frankfurt, Goethe University, 60590 Frankfurt, Germany; ^4^Department of Urology and Pediatric Urology, University Hospital Frankfurt, Goethe University, 60590 Frankfurt, Germany

## Abstract

*Background and Purpose.* Leukocyte migration into alveolar space plays a critical role in pulmonary inflammation resulting in lung injury. Acute ethanol (EtOH) exposure exerts anti-inflammatory effects. The clinical use of EtOH is critical due to its side effects. Here, we compared effects of EtOH and ethyl pyruvate (EtP) on neutrophil adhesion and activation of cultured alveolar epithelial cells (A549). *Experimental Approach.* Time course and dose-dependent release of interleukin- (IL-) 6 and IL-8 from A549 were measured after pretreatment of A549 with EtP (2.5–10 mM), sodium pyruvate (NaP, 10 mM), or EtOH (85–170 mM), and subsequent lipopolysaccharide or IL-1beta stimulation. Neutrophil adhesion to pretreated and stimulated A549 monolayers and CD54 surface expression were determined. *Key Results.* Treating A549 with EtOH or EtP reduced substantially the cytokine-induced release of IL-8 and IL-6. EtOH and EtP (but not NaP) reduced the adhesion of neutrophils to monolayers in a dose- and time-dependent fashion. CD54 expression on A549 decreased after EtOH or EtP treatment before IL-1beta stimulation. *Conclusions and Implications*. EtP reduces secretory and adhesive potential of lung epithelial cells under inflammatory conditions. These findings suggest EtP as a potential treatment alternative that mimics the anti-inflammatory effects of EtOH in early inflammatory response in lungs.

## 1. Introduction

Alcohol (ethanol), a well described immunomodulatory drug, exerts adverse and inconsistent effects on the inflammatory response depending of its either acute or chronic use as well as the dose. The pathogenesis of alcohol consumption is an important risk factor for several negative clinical outcomes. It has been associated with one-third of all traumatic injury deaths each year [1, 2]. Intoxicated trauma patients are at higher risk to gain infectious complications during their clinical course such as pneumonia, sepsis, and multiple organ failure (MOF) [3–9]. Therefore, the recovery after trauma or burn injury is prolonged [10]. In contrast, other studies report divergent results showing that acute alcohol intoxication does not affect the outcome and is even associated with decreased 24 h mortality after trauma compared to patients with chronic liver damage [11]. Chronic alcohol intake is linked to an increased proinflammatory cytokine response [12], whereas an acute or low-dose alcohol intake exerts anti-inflammatory effects [13, 14]. The impairment of host defense by alcohol and subsequent susceptibility to infections is associated with decreased polymorphonuclear neutrophil (PMN) migration as well as adherence to endothelial cells and reduced secretory rate of proinflammatory interleukin (IL)-8 [15]. On the other hand, moderate alcohol consumption is associated with decreased risk of cardiovascular disease events, such as myocardial infarction or stroke as well as lower incidence of admission coagulopathy in severe traumatic brain injury patients [16–18]. All these findings indicate a dose- and time-dependent influence of alcohol on the host immunity but also its strong therapeutic potential in acute inflammatory conditions. However, its practical application due to its entry into the CNS is limited in clinical settings.

Ethyl pyruvate (EtP), formed from pyruvate and ethanol, is a stable, well-tolerated compound that exerts similar antioxidant and anti-inflammatory effects as pyruvate [19, 20]. Ethyl pyruvate was found to protect from lipopolysaccharide- (LPS-) induced white matter injury in the developing rat brain [21], diminished the inflammatory response after LPS infusion* in vivo* [22], protected against ventilation-induced neutrophil infiltration and oxidative stress [23], and attenuated the hepatic injury severity in animals with severe acute pancreatitis [24]. Moreover, treatment with EtP even until 24 h after the onset of sepsis conferred beneficial effects* in vivo* [25]. Due to its good stability, the lack of side effects, the wide therapeutic window, and apparently no signs of intoxication on its use, EtP may be clinically useful for the treatment of acute inflammatory conditions.

Thus, we exposed immortalized human alveolar epithelial cells A549 to EtOH or EtP and to well-established proinflammatory agents LPS or IL-1beta to stimulate the cells. We evaluated whether EtP confers similar beneficial effects as EtOH in time- and dose-dependent manner in “inflamed” lung culture.

## 2. Material and Methods

### 2.1. Cell Culture

Human lung adenocarcinoma cell line A549 was purchased from Cell Lines Services (Heidelberg, Germany). The cells were cultured at 37°C under 5% CO_2_ in RPMI-1640 medium (Seromed, Berlin, Germany) supplemented with 10% heat-inactivated fetal calf serum (FCS), 100 IU/mL penicillin and 100 *μ*g/mL streptomycin (Gibco, Karlsruhe, Germany) and 20 mM HEPES buffer (Sigma, Steinheim, Germany). The culture media were changed every 2 or 3 days. The cell viability after stimulation with the various substances was assessed by the measurement of the cytoplasmic enzyme lactate dehydrogenase (LDH, Cytotoxicity Detection Kit, Roche, Penzberg, Germany) as described below.

The isolation of blood neutrophils (PMN) from healthy volunteers was in accordance with the Declaration of Helsinki and approved by the Institutional Ethics Committee of the Goethe University. All enrolled subjects gave informed consent themselves in accordance with ethical standards. PMN were isolated by density-gradient centrifugation (Polymorphprep, Nycomed, Oslo, Norway) according to manufacturer's instructions and as reported previously [26]. After isolation, PMN were cultured in RPMI-1640 medium as described above and their number as well as their viability was determined by the trypan blue exclusion assay. Only cell cultures with a purity of >95% were utilized for experimental use.

### 2.2. Cell Stimulation

The concentrations of EtOH, EtP, and NaP as well as IL-1beta and LPS are based on previous others' and own work to allow better comparison of data. EtOH was used at 85 and 170 mM (corresponding to 0.5–1 vol vol^−1^ percent, corresponding to 4–7.9 mg EtOH mL^−1^) as described previously [15, 27, 28]. Likewise, the concentrations of EtP (2.5 and 10 mM) and NaP (10 mM) were chosen from previous work [15, 29]. The cells were stimulated with either EtOH, EtP, or NaP for 1, 24, and 72 h to study acute and/or roughly chronic alcohol exposure effects. The schematic timeline of the experimental design for main experiments after dose and time course determination is shown in Figure 4.

The time and dose dependency of the secretory capacity of A549 cells was determined by the stimulation with either recombinant IL-1beta (0.1, 1, or 10 ng/mL, R&D Systems, Wiesbaden, Germany) or LPS from* E. coli* 0127:B8 (0.01, 0.1, 1 or 10 *μ*g/mL, Sigma) for 4, 8, 12, and 24 h. Due to the concentrations of secreted IL-6, IL-8, and TGF-beta we used IL-1beta at a dose of 1 ng/mL and LPS at 1 *μ*g/mL and stimulated A549 cells for 24 h to study the effects of EtOH and EtP.

### 2.3. Cell Viability

A549 cell viability was assessed by the measurement of the cytoplasmic LDH. In case of damaged plasma membrane the cells release LDH to the cell culture supernatant. The activity of LDH in supernatants collected from cells treated with EtOH, EtP, NaP, IL-1beta, and LPS in the dose- and time-dependent manner was determined enzymatically according to manufacturer's instruction (Cytotoxicity Detection Kit (LDH), Roche). A549 viability was >95% at the time and doses chosen for the treatment of the cells in each case. Moreover, no detachment of the cells was detected by microscopic evaluation of cell layers.

A trypan blue exclusion assay was used to determine the level of viability of PMN. Briefly, isolated PMN were stained with 0.4% trypan blue and about 100 cells were counted for each isolation. The mean percentage of viability was >99%.

### 2.4. Quantification of Cytokine Production

A549 cells were incubated with different doses of IL-1beta and LPS in a time course. At each time point culture supernatants were harvested and the concentrations of the cytokines IL-6, IL-8, and TGF-beta were determined by Quantikine Assays (R&D Systems) according to manufacturer's instructions. ELISA was performed using Infinite M200 microplate reader (Tecan, Männedorf, Switzerland).

To determine the effects of EtOH, EtP, and NaP on the cytokine production, A549 cells were preincubated with EtOH, EtP, or NaP for 1, 24, or 72 h prior the stimulation with IL-1beta or LPS for 24 h. Then, IL-6 and IL-8 were measured in culture supernatants as described above. To uncover the differences in cytokine release in these experiments, cytokine levels in percent relative to stimulated controls are expressed.

### 2.5. Ribonucleic Acid (RNA) Isolation, Quantitative Reverse-Transcription-Polymerase Chain Reaction (RT-PCR)

After preincubation with EtOH, EtP, or NaP for 1, 24, or 72 h and stimulation with IL-1beta or LPS for 24 h, total RNA of A549 was isolated using the RNeasy-system (Qiagen, Hilden, Germany) according to the manufacturer's instructions. The residual amounts of DNA remaining were removed using the RNase-Free DNase Set according to the manufacturer's instructions (Qiagen, Hilden, Germany). The RNA was stored immediately at −80°C. Quality and amount of the RNA were determined photometrically using the NanoDrop ND-1000 device (NanoDrop Technologies, Wilmington, DE, USA).

RNA was subsequently used for qRT-PCR. In brief, 100 ng of total RNA was reversely transcribed using the Affinity script QPCR-cDNA synthesis kit (Stratagene, La Jolla, CA, USA) following the manufacturer's instructions. To determine the mRNA expression of Hsp70, qRT-PCR was carried out on a Stratagene MX3005p QPCR system (Stratagene) using gene-specific primers for human HSPA4 (NM_002154, UniGene number: Hs.90093 Rn.9873, Cat number: PPH01188C) purchased from SABiosciences (SuperArray, Frederick, MD, USA). As reference gene, the expression of* GAPDH* with human GAPDH (NM 002046, UniGene number: Hs.592355, Cat number: PPH00150E; SABiosciences, SuperArray, Frederick, MD, USA) was measured. Sequences of these primers are not available. PCR reaction was set up with 1 × RT^2^ SYBR Green/Rox qPCR Master mix (SABiosciences) in a 25 *μ*L volume according to manufacturer's instructions. A two-step amplification protocol consisting of initial denaturation at 95°C for 10 min followed by 40 cycles with 15 s denaturation at 95°C and 60 s annealing/extension at 60°C was chosen. A melting-curve analysis was applied to control the specificity of amplification products.

Relative expression of target mRNA in each sample was calculated using the comparative threshold-cycle (CT) method (ΔCT method). In brief, the amount of target mRNA in each sample was normalized to the amount of* GAPDH* mRNA, to give ΔCT and then to a calibrator consisting of samples obtained from unstimulated but pretreated A549 cells. The relative mRNA expression of target genes is presented as percent change to unstimulated control calculated in relation to each unstimulated sample after normalization to* GAPDH*.

### 2.6. CD54 Surface Expression

After pretreatment with EtOH, EtP, and NaP and stimulation with IL-1beta and LPS, A549 cells were washed in PBS (0.5% bovine serum albumine, BSA) and then incubated with a fluorescein-conjugated mouse monoclonal antibody directed against ICAM-1/CD54 (BBIG-I1; R&D Sytems, Wiesbaden, Germany) for 60 min at 4°C. CD54 expression was measured by flow cytometry using FACS Calibur (BD Biosciences, Heidelberg, Germany, 1 × 10^4^ cells per scan) and expressed as mean fluorescence units (MFU). A mouse IgG1 fluorescein antibody (11711; R&D Systems) was used as an isotype control.

### 2.7. Monolayer Adhesion Assay

To analyze PMN adhesion to pretreated A549, A549 were transferred to 24-well multiplates (Falcon Primaria; Becton Dickinson, Heidelberg, Germany) in complete RPMI-1640 medium. When a confluency of ~80% was reached, A549 cells were preincubated with EtOH, EtP, or NaP for 1 h and stimulated with IL-1beta or LPS for 24 h. Then freshly isolated PMN (5 × 10^4^ cells/well) were carefully added to the A549 monolayer or to an empty plastic surface for 60 min. Subsequently, nonadherent PMN were washed off 3x using warmed (37°C) complete RPMI-1640 medium. The remaining PMN were fixed with 1% glutaraldehyde. Adherent PMN were counted in five different fields of a defined size (5 × 0.25 mm^2^) using a phase contrast microscope (×20 objective) and the mean cellular adhesion rate was calculated.

### 2.8. Statistical Analysis

All experiments were performed 3–6 times. Differences between groups were determined by Wilcoxon-Mann-Whitney* U*-test. A* P* value of less than 0.05 was considered significant. Data are given as mean ± standard error of the mean (s.e.m.). All statistical analyses were performed employing GraphPad Prism 5 (Graphpad Software, Inc., San Diego, CA).

## 3. Results

### 3.1. Measurement of the Secretory Potential of A549 Cells

In order to reveal the secretory potential as well as the time and dose response of A549 cells to proinflammatory mediators, A549 release of IL-8, TGF-beta, and IL-6 after either IL-1beta or LPS stimulation was evaluated.

#### 3.1.1. IL-8 Release

**Figure 1:**
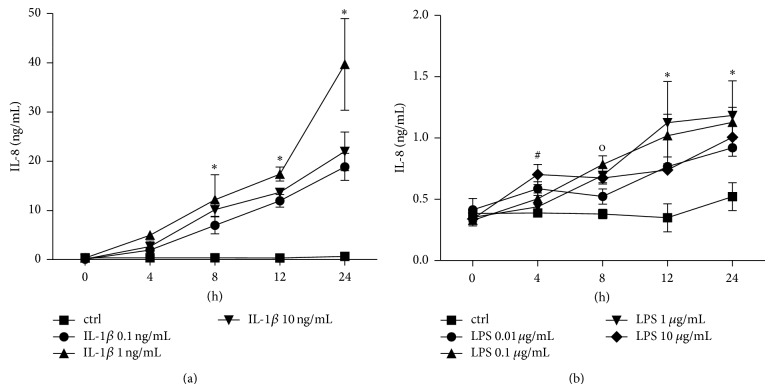
Interleukin (IL)-8 release from lung epithelial cells A549 after IL-1beta (a) or lipopolysaccharide (LPS (b)) stimulation. Cells were stimulated with IL-1beta or LPS in indicated concentrations for different intervals (incubation time indicated below the *x*-axis). After the incubation periods, supernatants were analyzed for IL-8 concentrations. The data are presented as means ± s.e.m. ^*^ *P* < 0.05, all groups versus corresponding control (ctrl); ^#^ *P* < 0.05, LPS 10 *μ*g/mL versus corresponding ctrl; ^o^ *P* < 0.05, all groups except LPS 0.01 *μ*g/mL versus corresponding ctrl.

IL-1beta and LPS induced a dose- and time-dependent release of IL-8 (Figure 1). IL-1beta in all used concentrations (0.1, 1 and 10 ng/mL) enhanced the IL-8 release continuously with the increasing incubation time (Figure 1(a)). The dose response curve peaked by 24 h of incubation with the 1 ng/mL IL-1beta stimulation dose from 0.67 ± 0.04 to 39.66 ± 9.33 ng/mL IL-8 (*P* < 0.05, Figure 1(a)). LPS enhanced slightly the IL-8 release depending on the dose, but after 24 h stimulation with 1 *μ*g/mL the dose response peaked to 1.18 ± 0.28 compared to 0.52 ± 0.11 ng/mL IL-8 in unstimulated samples after 24 h (*P* < 0.05, Figure 1(b)).

#### 3.1.2. TGF-Beta Release

**Figure 2:**
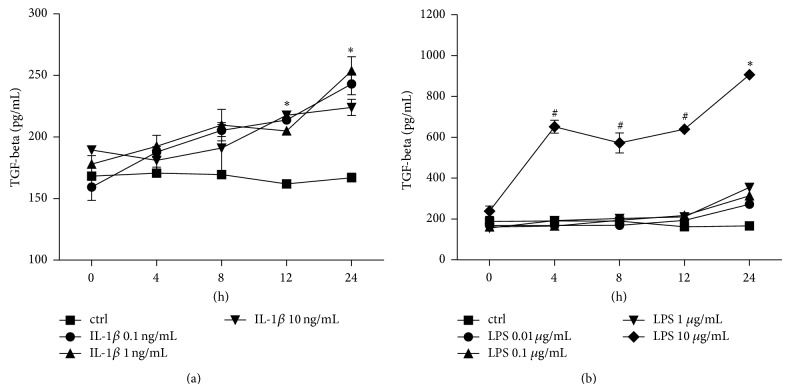
Transforming growth factor- (TGF-) beta release from lung epithelial cells A549 after IL-1beta (a) or lipopolysaccharide (LPS (b)) stimulation. Cells were stimulated with IL-1beta or LPS in indicated concentrations for different intervals (incubation time indicated below the *x*-axis). After the incubation periods, supernatants were analyzed for TGF-beta concentrations. The data are presented as means ± s.e.m. ^*^ *P* < 0.05, all groups versus corresponding control (ctrl); ^#^ *P* < 0.05, LPS 10 *μ*g/mL versus corresponding ctrl.

IL-1beta and LPS induced a dose- and time-dependent release of TGF-beta also (Figure 2). In each concentration (0.1, 1, and 10 ng/mL) IL-1beta enhanced the TGF-beta release with significant peaks after 24 h (Figure 2(a)). The highest response was observed after 24 h stimulation with 1 ng/mL IL-1beta increasing from 166.90 ± 3.66 in unstimulated ctrl to 253.70 ± 11.35 pg/mL TGF-beta (*P* < 0.05, Figure 2(a)). LPS enhanced the TGF-beta release at each time point at the highest dose (10 *μ*g/mL) with a strong peak after 24 h (905.90 ± 15.53 pg/mL TGF-beta, Figure 2(b)). Lower doses of LPS increased TGF-beta release only after 24 h incubation period, reaching the significant peak at 354.60 ± 17.32 pg/mL TGF-beta compared to unstimulated ctrl after stimulation with 1 *μ*g/mL LPS (*P* < 0.05, Figure 2(b)).

#### 3.1.3. IL-6 Release

**Figure 3:**
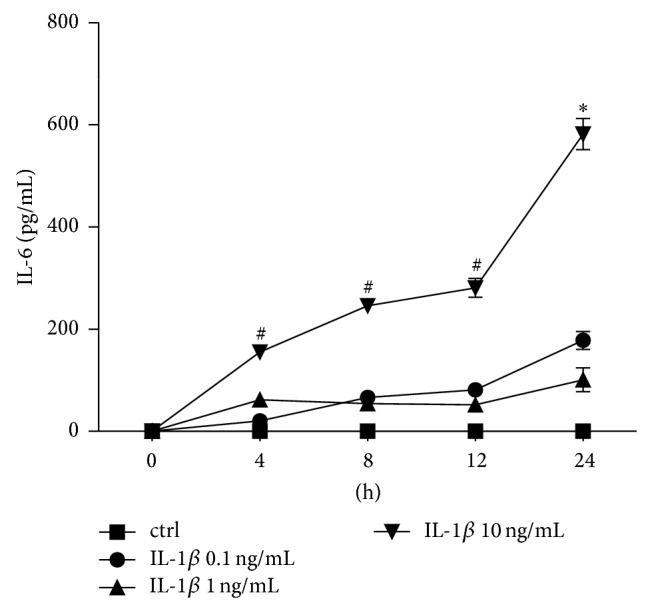
Interleukin (IL)-6 release from lung epithelial cells A549 after IL-1beta stimulation. Cells were stimulated with IL-1beta in indicated concentrations for different intervals (incubation time indicated below the *x*-axis). After the incubation periods, supernatants were analyzed for IL-6 concentrations. The data are presented as means ± s.e.m. ^*^ *P* < 0.05, all groups versus corresponding control (ctrl); ^#^ *P* < 0.05, IL-1beta 10 ng/mL versus corresponding ctrl.

After incubation of A549 cells with IL-1beta there was a significant IL-6 dose response to 10 ng/mL IL-1beta at each incubation period compared to unstimulated controls (*P* < 0.05, Figure 3). This dose response was not observed at lower doses of IL-1beta after 4, 8, or 12 h incubation. After 24 h incubation with lower doses of IL-1beta, enhanced IL-6 release was detected in all stimulated samples compared to controls (Figure 3). LPS stimulation did not markedly alterate the IL-6 release in A549 cells in this experimental setting (data not shown).

### 3.2. Cytokine Production After EtOH or EtP Treatment

**Figure 4:**
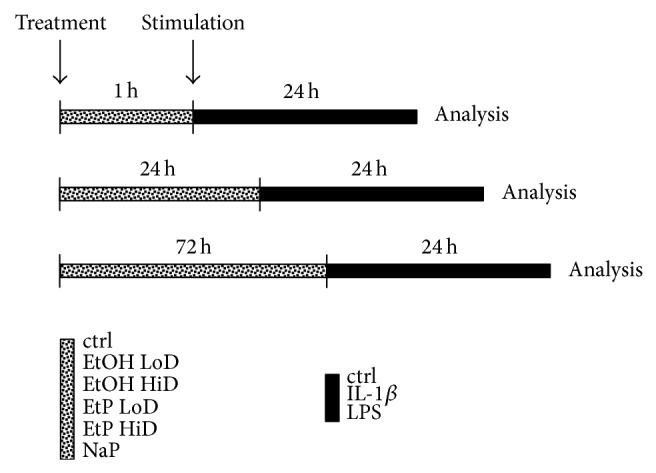
Schematic timeline of the experimental design. Cells were treated with EtOH (low dose, LoD = 85 mM and high dose, HiD = 170 mM), EtP (LoD = 2.5 mM and HiD = 10 mM), or NaP (10 mM) for 1 h, 24 h, or 72 h and then stimulated with IL-1beta (1 ng/mL) or LPS (1 *μ*g/mL) for 24 h. After the incubation periods, the analyses were performed.

Previously it has been reported that short incubation with EtP reduced the release of IL-8 in both stimulated human endothelial and epithelial cells [15, 30]. Here, we evaluated the effects on the secretory potential of proinflammatory cytokines IL-8 and IL-6 by A549 cells after their pretreatment with EtOH, EtP, or NaP for 1, 24, or 72 h and subsequent stimulation with IL-1beta or LPS for 24 h (Figure 4).

#### 3.2.1. IL-8 Release

**Figure 5:**
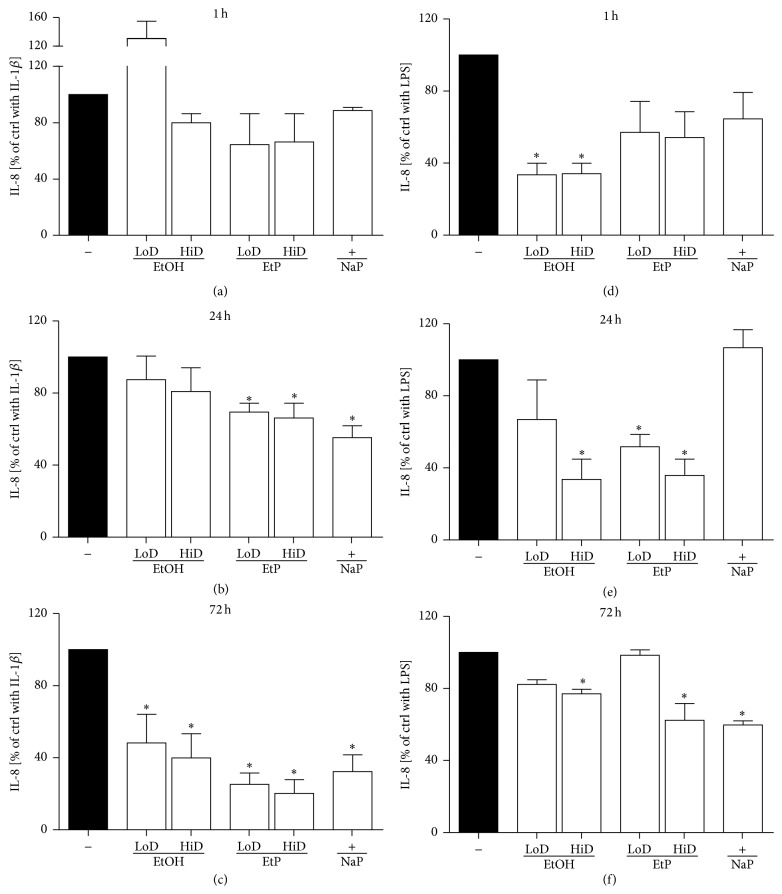
Effects of ethanol (EtOH), ethyl pyruvate (EtP), or sodium pyruvate (NaP) on interleukin (IL)-8 release from lung epithelial cells A549 after IL-1beta ((a)–(c)) or lipopolysaccharide (LPS (d)–(f)) stimulation. Cells were treated with EtOH (low dose, LoD = 85 mM and high dose, HiD = 170 mM), EtP (LoD = 2.5 mM and HiD = 10 mM), or NaP (10 mM) for 1 h ((a) and (d)), 24 h ((b) and (e)), or 72 h ((c) and (f)) and then stimulated with IL-1beta (1 ng/mL) or LPS (1 *μ*g/mL) for 24 h. After the incubation periods, supernatants were analyzed for IL-8 concentrations (given as % of cells stimulated with agonists only, control, ctrl). The data are presented as means ± s.e.m. ^*^ *P* < 0.05 versus ctrl.

In A549 cells, IL-1beta (1 ng/mL) or LPS (1 *μ*g/mL) caused a significant increase in IL-8 release after 24 h as shown in Figure 1. Treatment with EtOH for 1 or 24 h did not change the IL-8 release, whereas the treatment with EtOH for 72 h significantly decreased the IL-8 release to 48% at low dose (85 mM) and 40% at high dose (170 mM) EtOH compared to IL-1beta-unstimulated samples (*P* < 0.05, Figures 5(a)–5(c)). IL-8 release from LPS-stimulated A549 cells was diminished by both doses of EtOH already after 1 h treatment compared to untreated LPS-stimulated ctrl (34% versus 100%, *P* < 0.05, Figure 5(a)). After 24 h only low dose EtOH diminished the IL-8 release to 34% significantly (*P* < 0.05, Figure 5(b)). 72 h treatment with EtOH reduced markedly IL-8 release at low dose EtOH but this reduction was significant only after high dose EtOH treatment.

Treatment with both doses of EtP caused a significant reduction of IL-1beta induced IL-8 release compared to stimulated untreated ctrl only after 24 and 72 h (24 h: 2.5 mM EtP, 69% IL-8 release, 10 mM EtP, 66% IL-8 release; 72 h: 2.5 mM EtP, 25% and 10 mM EtP, 20% IL-8 release, respectively, *P* < 0.05, Figures 5(a)–5(c)). After LPS-stimulation, treatment with EtP for 1 h in both doses did not confer significant changes in IL-8 release (Figure 5(d)). Treatment with EtP for 24 h dose-dependently reduced IL-8 release to 52% and 36%, respectively (*P* < 0.05, Figure 5(e)). After 72 h, only 10 mM EtP treatment diminished significantly the IL-8 release to 62% (*P* < 0.05, Figure 5(f)). NaP reduced LPS-stimulated IL-8 release significantly after 72 h treatment (Figures 5(d)–5(f)).

#### 3.2.2. IL-6 Release

**Figure 6:**
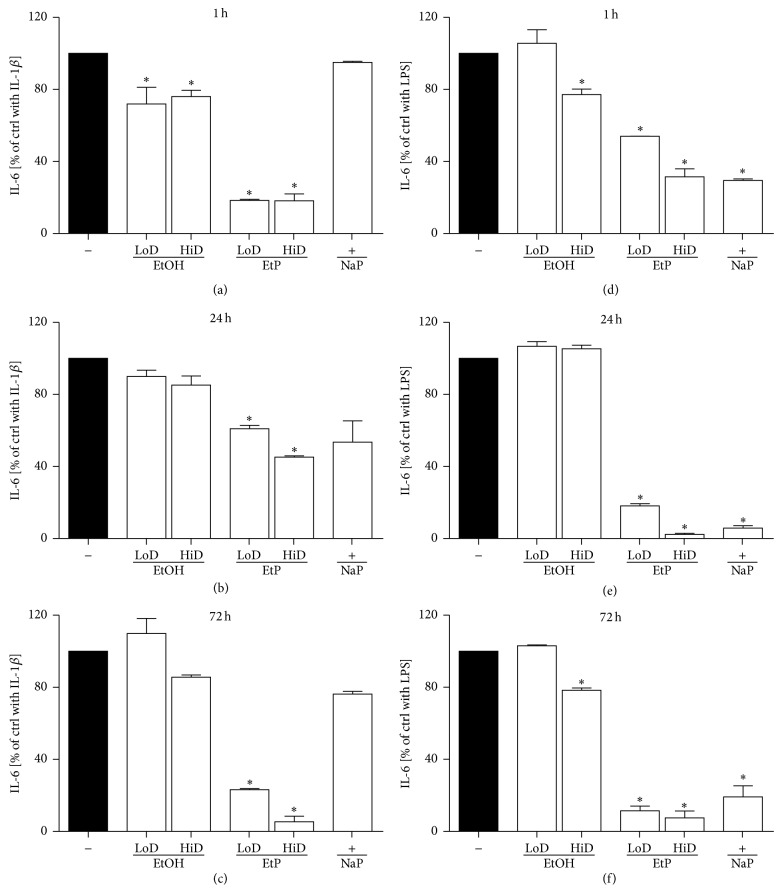
Effects of ethanol (EtOH), ethyl pyruvate (EtP), or sodium pyruvate (NaP) on interleukin (IL)-6 release from lung epithelial cells A549 after IL-1beta ((a)–(c)) or lipopolysaccharide (LPS (d)–(f)) stimulation. Cells were treated with EtOH (low dose, LoD = 85 mM and high dose, HiD = 170 mM), EtP (LoD = 2.5 mM, HiD = 10 mM), or NaP (10 mM) for 1 h ((a) and (d)), 24 h ((b) and (e)), or 72 h ((c) and (f)) and then stimulated with IL-1beta (1 ng/mL) or LPS (1 *μ*g/mL) for 24 h. After the incubation periods, supernatants were analyzed for IL-6 concentrations (given as % of cells stimulated with agonists only, control, ctrl). The data are presented as means ± s.e.m. ^*^ *P* < 0.05 versus ctrl.

IL-1 beta induced increase of IL-6 release was significantly reduced by both EtOH doses to 72% (85 mM) and 76% (170 mM), respectively at 1 h treatment as compared to untreated stimulated ctrl (*P* < 0.05, Figure 6(a)). 24 h and 72 h treatment with EtOH did not change the IL-6 release markedly (Figures 6(b) and 6(c)). Treatment with 85 mM EtOH prior to LPS stimulation did not alter the IL-6 release at any incubation period. High dose EtOH (170 mM) reduced significantly the IL-6 release to 77% after 1 h and to 78% after 72 h pretreatment prior to LPS stimulation compared to untreated stimulated ctrl (*P* < 0.05, Figures 6(d) and 6(f)). At 24 h preincubation EtOH did not confer any changes in IL-6 release (Figure 6(e)).

EtP significantly and dose-dependently inhibited the IL-1beta-stimulated IL-6 release to 18%, 61%, and 23% at low dose (2.5 mM) and to 18%, 45%, and 5% at high dose (10 mM) after 1, 24, and 72 h pretreatment, respectively (*P* < 0.05, Figures 6(d)–6(f)). Treatment with 2.5 and 10 mM EtP diminished significantly the IL-6 release to 54% and 32% (1 h), 18% and 2% (24 h), and 11% and 8% (72 h), respectively, when compared to untreated stimulated ctrl (*P* < 0.05, Figures 6(d)–6(f)). Furthermore, NaP conferred significant reduction of LPS-induced IL-6 release at any incubation period (*P* < 0.05, Figures 6(d)–6(f)).

### 3.3. Hsp70 Gene Expression

**Figure 7:**
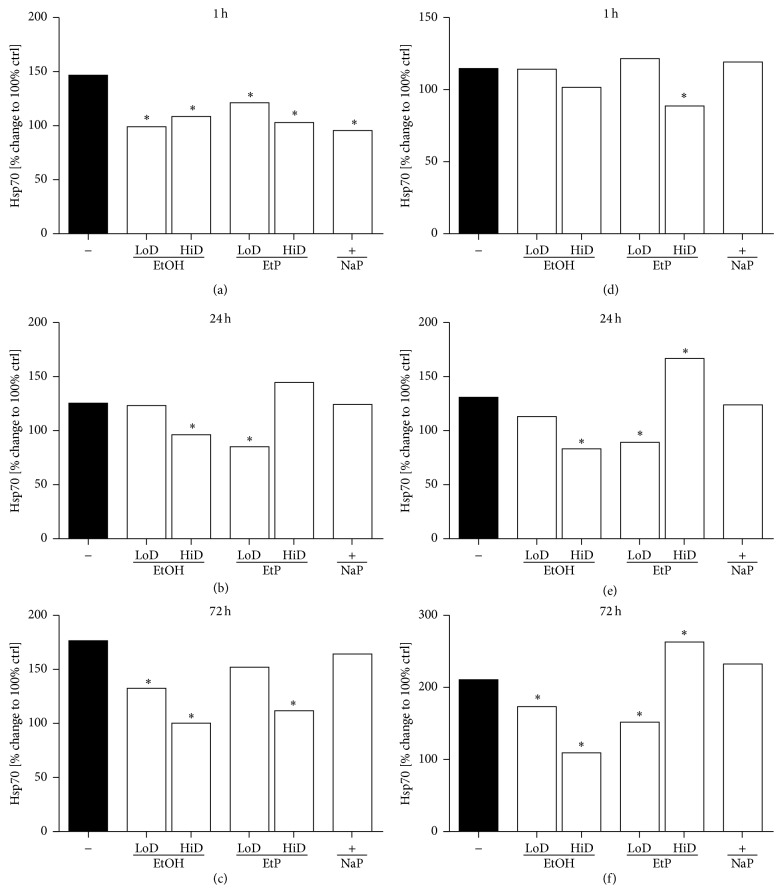
Effects of ethanol (EtOH), ethyl pyruvate (EtP), or sodium pyruvate (NaP) on heat-shock protein (hsp70) gene expression in lung epithelial cells A549 after IL-1beta ((a)–(c)) or lipopolysaccharide (LPS (d)–(f)) stimulation. Cells were treated with EtOH (low dose, LoD = 85 mM and high dose, HiD = 170 mM), EtP (LoD = 2.5 mM, HiD = 10 mM), or NaP (10 mM) for 1 h ((a) and (d)), 24 h ((b) and (e)), or 72 h ((c) and (f)) and then stimulated with IL-1beta (1 ng/mL) or LPS (1 *μ*g/mL) for 24 h. After normalization to GAPDH expression, gene expression was measured as % change compared to hsp70 expression in cells stimulated with agonists only (control, ctrl, as 100%). ^*^ *P* < 0.05 versus untreated stimulated ctrl.

The real-time PCR showed significantly increased Hsp70 expression after IL-1beta and LPS stimulation compared to unstimulated cells collected at each time point 1 h (146% and 115%), 24 h (125% and 131%), and 72 h (176% and 210%, resp., *P* < 0.05 versus unstimulated cells, Figure 7). A549 cells treated with 85 mM or 180 mM EtOH for 1 h and then stimulated with IL-1beta expressed significantly less Hsp70 (99% and 109%, resp.; *P* < 0.05, Figure 7(a)). Low dose EtOH pretreatment (85 mM) for 24 h hours did not cause changes in Hsp70 gene expression, whereas high dose EtOH (170 mM) reduced markedly the gene expression compared to stimulated untreated cells (*P* < 0.05, Figure 7(b)). Both EtOH doses diminished significantly the Hsp70 expression after 72 h pretreatment to 133% (85 mM EtOH) and 100% (170 mM EtOH) compared to untreated stimulated ctrl (*P* < 0.05, Figure 7(c)). In LPS stimulated A549 cells, EtOH reduced significantly the Hsp70 expression after 24 h pretreatment in high dose (170 mM) to 83% and in both doses to 173% (85 mM) and 110% (170 mM) after 72 h pretreatment compared to stimulated untreated ctrl (*P* < 0.05, Figures 7(e) and 7(f)).

Treatment with both doses of EtP caused a significant reduction of Hsp70 expression in IL-1beta stimulated cells after 1 h pretreatment (2.5 mM: 121% and 10 mM: 103%, resp.) compared to untreated stimulated ctrl (146%, *P* < 0.05, Figure 7(a)). The IL-1beta induced increase in Hsp70 expression was reduced to 85% in cells treated with low dose EtP (2.5 mM, *P* < 0.05), whereas high dose EtP (10 mM) conferred no changes (Figure 7(b)). After 72 h only pretreatment with high dose EtP diminished markedly the Hsp70 expression to 112% (*P* < 0.05, Figure 7(c)). In LPS stimulated A549 cells, high dose EtP (10 mM) for 1 h and low dose EtP for 24 h and 72 h diminished strongly the Hsp70 expression to 89%, 89%, and 152%, respectively, when compared to corresponding untreated stimulated ctrl (*P* < 0.05, Figures 7(d) and 7(e)). High dose EtP (10 mM) for 24 h or 72 h increased significantly the Hsp70 expression to 167% and 263%, respectively, compared to corresponding untreated stimulated ctrl (*P* < 0.05, Figures 7(e) and 7(f)). NaP reduced the Hsp70 expression significantly only at 1 h pretreatment (96%, *P* < 0.05, Figure 7) in IL-1beta stimulated A549 cells.

### 3.4. CD54 Adhesion Protein Expression

**Figure 8:**
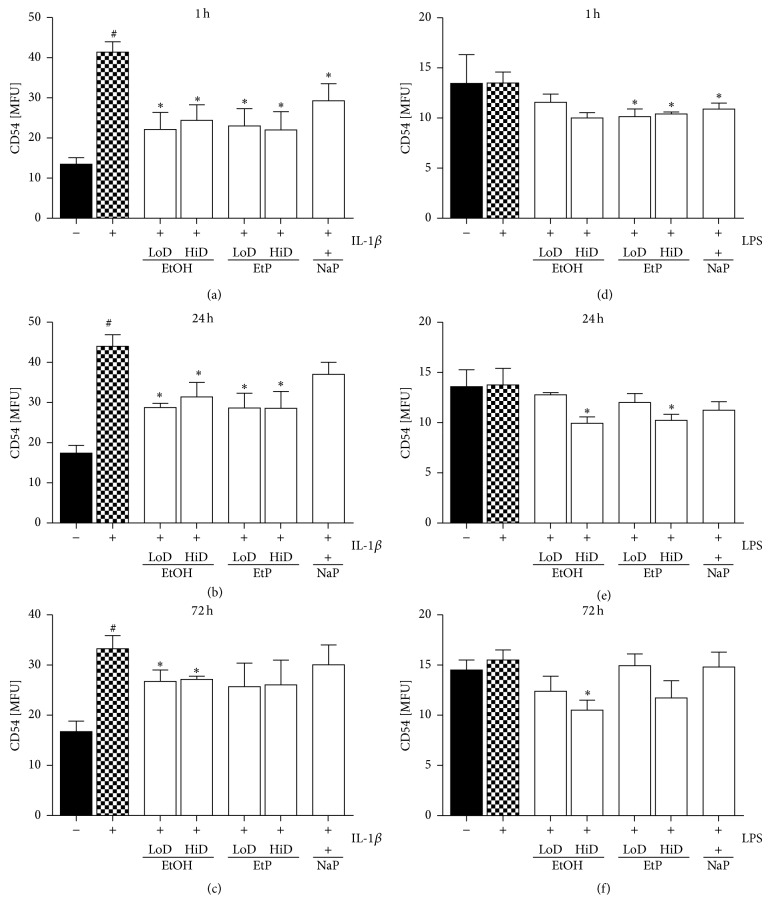
Effects of ethanol (EtOH), ethyl pyruvate (EtP), or sodium pyruvate (NaP) on the surface expression of CD54 in lung epithelial cells A549 after IL-1beta ((a)–(c)) or lipopolysaccharide (LPS (d)–(f)) stimulation. Cells were treated with EtOH (low dose, LoD = 85 mM and high dose, HiD = 170 mM), EtP (LoD = 2.5 mM, HiD = 10 mM), or NaP (10 mM) for 1 h ((a) and (d)), 24 h ((b) and (e)), or 72 h ((c) and (f)) and then stimulated with IL-1beta (1 ng/mL) or LPS (1 *μ*g/mL) for 24 h. After the incubation periods, CD54 expression was evaluated (given as mean fluorescence unit, MFU). The data are presented as means ± s.e.m. ^*^ *P* < 0.05 versus untreated but stimulated control, ctrl; ^#^ *P* < 0.05 versus untreated and unstimulated cells.

IL-1beta stimulation of A549 cells induced a significant increase in surface CD54 protein expression compared to unstimulated ctrl (*P* < 0.05, Figures 8(a)–8(c)). Treatment with EtOH for 1 h, 24 h, or 72 h reduced strongly CD54 protein expression in both, low (85 mM), and high dose (170 mM) to 22 and 24 (1 h), 29 and 31 (24 h), or 27 MFU (both, 72 h) compared to stimulated untreated ctrl (41, 44, 33 MFU, resp.; *P* < 0.05, Figures 8(a)–8(c)). LPS stimulation did not change the CD54 expression on A549 cells markedly. However, 170 mM EtOH treatment reduced CD54 expression after 1 h, 24 h, or 72 h pretreatment to 10 MFU compared to untreated stimulated ctrl (each, *P* < 0.05, Figures 8(d) and 8(e)). CD54 protein expression on IL-1beta stimulated A549 cells was diminished by both low and high dose of EtP after 1 h as well as 24 h pretreatment compared to untreated IL-1beta stimulated ctrl (23 and 22 versus 41 MFU, as well as 29 each versus 44 MFU; *P* < 0.05, Figures 8(a) and 8(b)). After 72 h EtP did not change CD54 expression. In LPS stimulated A549 cells, EtP reduced the CD54 expression to 10 MFU when used in both doses for 1 h compared to untreated LPS stimulated ctrl (*P* < 0.05, Figure 8(a)). After 24 h only high dose EtP reduced significantly the CD54 expression from 14 to 10 MFU (*P* < 0.05, Figure 8(b)). Other incubation times with EtP as well as NaP treatment did not show significant changes (Figures 8(d)–8(f)).

### 3.5. PMN Adherence

**Figure 9:**
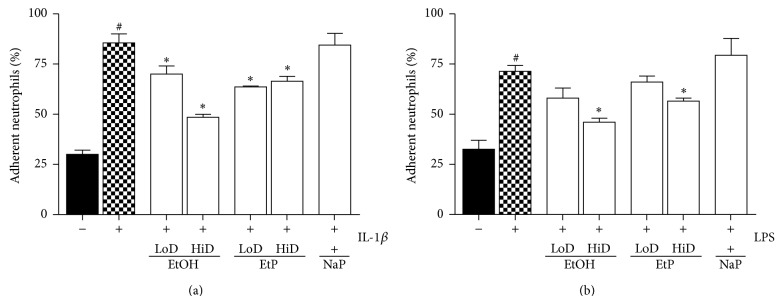
Effects of ethanol (EtOH), ethyl pyruvate (EtP), or sodium pyruvate (NaP) on the adhesiveness of neutrophils to lung epithelial cells A549 after IL-1beta (a) or lipopolysaccharide (LPS) stimulation. Cells were treated with EtOH (low dose, LoD = 85 mM and high dose, HiD = 170 mM), EtP (LoD = 2.5 mM and HiD = 10 mM), or NaP (10 mM) for 1 h and then stimulated with IL-1beta (1 ng/mL) or LPS (1 *μ*g/mL) for 24 h. After the incubation periods, neutrophils were added and the adhesion capacity after 30 minutes was analyzed (given as % of total neutrophils). The data are presented as means ± s.e.m. ^*^ *P* < 0.05 versus untreated but stimulated control, ctrl; ^#^ *P* < 0.05 versus untreated and unstimulated cells.

The adhesion capacity of PMN to A549 monolayer significantly enhanced from 30% to 86% after IL-1beta stimulation and from 33% to 71% after LPS stimulation of A549 cells (*P* < 0.05, Figures 9(a) and 9(b)). Treatment of A549 monolayers with EtOH (85 mM and 170 mM) for 1 h significantly reduced the PMN adhesion to 70% and 49% compared to PMN adherence to untreated IL-1beta stimulated A549 cells (*P* < 0.05, Figure 9(a)). In LPS stimulated A549 cells, reduced PMN adherence was observed only in samples pretreated with high dose EtOH (46%, *P* < 0.05, Figure 9(b)). EtP pretreatment of IL-beta stimulated cells diminished significantly the PMN adhesion to 64% (85 mM EtOH) and 66% (170 mM EtOH), respectively (*P* < 0.05, Figure 9(a)). In LPS stimulated samples, only high dose EtP (10 mM) for 1 h reduced markedly the adhesion of PMN to 59% (*P* < 0.05, Figure 9(b)). Treatment of A549 monolayers with NaP did not alter the PMN adherence (Figure 9).

## 4. Discussion

In the present study, we evaluated effects of acute and prolonged alcohol as well as ethyl pyruvate use on the proinflammatory responses of human lung epithelial cells to IL-1beta and LPS stimulation (Figure 4). Exposure to EtOH or EtP suppressed these responses to IL-1beta or LPS stimulation. Both substances inhibited in a dose- and time-dependent manner the IL-1beta as well as LPS-induced IL-8 and IL-6 release. These effects were accompanied by modified induction of Hsp70 in response to stimuli demonstrating a rather decreased hsp70 induction by EtOH or EtP after IL-1beta stimulation during the whole time course. However, after LPS stimulation EtOH delivered similar results as those in IL-1beta stimulated samples, whereas EtP in low dose decreased hsp70 induction with a clear tendency to increase it when applied at high dose in prolonged incubation model. The dampened cytokine release by EtOH or EtP as well as hsp70 expression were accompanied by the inhibited surface expression of CD54 especially in acute incubation conditions. The adherence of neutrophils to pretreated and IL-1beta or LPS stimulated lung epithelial cells was decreased by both EtOH and EtP.

Previous studies have demonstrated the immunomodulatory potential of EtOH consumption in various models of inflammation. Although the effects of chronic alcohol consumption are associated with increased proinflammatory cytokine response and these effects appear unfavourable, its moderate or acute intake has several favourable and anti-inflammatory effects [12–14, 31–33]. Lipopolysaccharide (LPS), known as endotoxin and proinflammatory cytokines, such as IL-1beta, IL-6, and IL-8, have been identified as important contributors to the pathogenesis of organ injury including lung injury in models of acute inflammation [34–37]. Acute alcohol intake reduces LPS-induced IL-6 production from macrophages in a time- and dose-dependent manner [38]. Moreover, Johansson et al. [15] reported that acute treatment of human umbilical vein cells (HUVEC) with the alcohol dose that was used in our study and subsequent stimulation of cells with LPS or IL-1beta resulted in decreased release of IL-8 and granulocyte colony-stimulating factor (G-CSF), respectively [15]. The present study of IL-8 and IL-6 release from stimulated lung epithelial cells demonstrated that effects of alcohol differed depending on the used stimulus, either IL-1beta or LPS (Figures 5 and 6). In IL-1beta stimulated cells, both alcohol doses had anti-inflammatory effects concerning the IL-8 release only when used for a prolonged (72 h) incubation period, whereas similar effects concerning IL-6 release were observed only under acute incubatory conditions (1 h). LPS-induced IL-8 and IL-6 releases were prevented predominantly by high dose alcohol in both acute and prolonged stimulation model. Given these findings, it is likely that high dose alcohol exerts rather potent anti-inflammatory effects than low dose alcohol. Other studies have confirmed that the viability of human endothelial cells is above 95% after their pretreatment with the high dose alcohol (170 mM) used in our study also and subsequent stimulation with IL-1beta for 24 h [15, 27]. Mice treated with alcohol and then challenged intraperitoneally with nonpathogenic* E. coli* demonstrated suppressed production of most proinflammatory cytokines [39]. In that study, the authors demonstrate that alcohol treatment had different effects on different cytokines, maybe due to their induction by different receptors like TLRs [39]. Neutrophils and epithelial cells represent the first line of defense in inflammatory conditions. Lung epithelial cells play a decisive role in the pulmonary innate immune response [40, 41]. However, neutrophils are required for the host defense but in a large line of studies it has been demonstrated that their inhibited delivery to inflammatory sites enhances organ integrity. Previously, we have demonstrated that the reduced expression of hepatic CD54 by acute alcohol application was associated with decreased hepatic neutrophil infiltration in an acute model of inflammation [32]. In line with these findings, Jonsson and Palmblad [27] demonstrated increased CD54 expression on HUVEC after IL-1beta or LPS stimulation and enhanced neutrophil adhesiveness to these cells [27]. In the same study, alcohol inhibited the LPS-induced adhesion of neutrophils to stimulated HUVEC but it did not affect the CD54 expression [27]. Here, alcohol inhibited moderately the CD54 expression at each incubation period demonstrating stronger effects when used in higher dose (Figure 8). Acute alcohol exposure of lung epithelial cells prior to their stimulation with IL-1beta or LPS was associated with decreased neutrophil adhesion capacity (Figure 9). The analysis of the* hsp70* gene expression to uncover the cellular stress state in pretreated and stimulated lung epithelial cells demonstrated predominantly reduced hsp70 at high dose alcohol (Figure 7). The induction of hsp70 in response to stressors is thought to prevent cytotoxicity and cell death [42]. Low concentrations of alcohol increase the hsp70 expression in intestinal cells [43]. In chronic alcohol use the immunoproteasome dysformation and dysfunction are parallel by increased hsp70 supposing to compensate the unfolding/docking of misfolded proteins by the proteasome [44]. Interestingly, Collins et al. [45] reported that significant elevations of hsp70 in rat brain cultures were observed after 6 days of moderate ethanol exposure but not at 4 days [45]. Our results may be explained by the short culture period; however, further experiments on this are required. Other and our studies confirm the dose- and time-dependent influence of alcohol on host immunity that can be beneficial in acute inflammatory conditions. However, due to its entry into the CNS its practical application in the clinical setting as potential therapy is not suitable. Moreover, there are no randomized prospective clinical trials to evaluate alcohol effects in dose- and time-dependent manner. With regard to even its moderate use the risk of addiction is given. Therefore, even acute and low dose alcohol therapeutic application does not seem encouraging. For many other reasons, other treatment options with similar effects but lack of adverse events would be beneficial. Ethyl pyruvate was found to be safe, well-tolerated, and promising as an anti-inflammatory drug [19]. In our study, the direct comparison of alcohol with EtP revealed higher potential of EtP even when used in the lower dose to diminish the proinflammatory cytokine release from lung epithelial cells after stimulation independently from the incubation period (Figures 5 and 6). Our findings are in line with previously published data by Johansson and Palmblad [30] that confirm the anti-inflammatory potential of EtP [30]. Here, we demonstrate that the application of EtP leads to beneficial effects independently from the incubation period and that these effects are stronger than those induced by alcohol. Nonetheless, while these findings are quite different, the effects concerning CD54 expression and neutrophil adhesion capacity to preincubated and stimulated lung epithelial cells are rather similar between EtP and alcohol. EtP decreases the CD54 expression predominantly at early incubation periods as alcohol does (Figure 8). Similarly, both alcohol and EtP reduce the neutrophil adhesion rates to lung epithelial cells (Figure 9). As we described previously, alcohol reduced hsp70 expression. EtP exerts similar effects with the exception of the treatment for prolonged incubation period with EtP before LPS stimulation (Figure 7). Here, we observed even significant increase in hsp70 indicating ongoing early cellular protection mechanisms. Therefore, due to even more consistent anti-inflammatory potential of EtP compared to alcohol independently from the stimuli used, EtP might represent a useful therapeutic tool that has to be investigated in further studies.

With regard to sodium pyruvate treatment, we did not gain consistent results. While NaP reduces to some extent the IL-8 and IL-6 release from lung epithelial cells, in comparison with EtP this effect is rather weak. On the other hand, NaP was nearly without any effects concerning CD54 expression, neutrophil adhesion, and hsp70 expression (Figures 8 and 9). These findings suggest that the pyruvate moiety of both molecules is as well essential for the cytokine release, whereas the ethyl moiety of the EtP or the alcohol molecule seems essential for the functional such as adhesion mechanisms. These results suggest also EtP as the most potent anti-inflammatory drug in our experimental setting. However, the study findings are clearly limited by the pretreatment conditions.

Taken together, we demonstrated a reduction of the proinflammatory cytokine release from stimulated lung epithelial cells by alcohol as well as ethyl pyruvate. Furthermore, reduced adhesion molecule surface expression as well as the adhesion capacity of neutrophils is decreased by alcohol and ethyl pyruvate. Due to its good stability and apparently wide therapeutic window, ethyl pyruvate should be tested in a posttreatment experimental and clinical setting.
